# Beyond Research Ethics: Dialogues in Neuro-ICT Research

**DOI:** 10.3389/fnhum.2019.00105

**Published:** 2019-03-29

**Authors:** Bernd Carsten Stahl, Simisola Akintoye, B. Tyr Fothergill, Manuel Guerrero, Will Knight, Inga Ulnicane

**Affiliations:** ^1^Centre for Computing and Social Responsibility, De Montfort University, Leicester, United Kingdom; ^2^Leicester De Montfort Law School, De Montfort University, Leicester, United Kingdom; ^3^Department of Neurobiology, Care Sciences and Society, Division of Neurogeriatrics, Karolinska Institutet, Solna, Sweden; ^4^Centre for Research Ethics and Bioethics, Uppsala University, Uppsala, Sweden; ^5^Department of Bioethics and Medical Humanities, University of Chile, Santiago, Chile

**Keywords:** ethics, IRB, review, ethics support, discourse, human brain project, discourse ethics

## Abstract

The increasing use of information and communication technologies (ICTs) to help facilitate neuroscience adds a new level of complexity to the question of how ethical issues of such research can be identified and addressed. Current research ethics practice, based on ethics reviews by institutional review boards (IRB) and underpinned by ethical principlism, has been widely criticized. In this article, we develop an alternative way of approaching ethics in neuro-ICT research, based on discourse ethics, which implements Responsible Research and Innovation (RRI) through dialogues. We draw on our work in Ethics Support, using the Human Brain Project (HBP) as empirical evidence of the viability of this approach.

## Introduction

Biomedical research, including research in neuroscience, is fundamentally driven by the conviction that this type of research is morally good and desirable. The large amounts of public funding available for this type of research are provided because of the hope that the research outcomes will contribute to the public good and offer a better understanding of the brain. At the same time, such research can raise significant ethical concerns. Traditionally, these are linked to the nature of the research, e.g., invasive research on humans and other animals.

At present, one can observe a continuing increase in the emphasis on the use of information and communication technologies (ICTs) for the collection and analysis of ever-larger neuroscientific datasets. Such work, which we frame as “neuro-ICT” draws on different disciplines including neuroscience and computer science to further our understanding of the brain. This type of work can raise additional ethical and social questions that can originate from either of the parent disciplines or from interdisciplinary applications.

Addressing these ethical and social concerns appropriately is a precondition for gaining and maintaining institutional approval and political support, which is a requirement for both continued funding and successful publication of the eventual findings. Given past scandals involving biomedical research, a sensitive way of dealing with ethics is a requirement for retaining the public trust and the societal “license to operate.”

Neuroscientists, in the tradition of biomedical sciences, develop an understanding of the ethics requirement during their socialization into the field and are normally familiar with the standard requirements of gaining ethics approval. Many of the ethical issues that neuroscientists encounter are well established and procedures for dealing with them are well known. This is true for many aspects of human subject research as well as animal research. Computer scientists, engineers and other technicians and physical scientists tend to have a qualitatively different exposure to and experience of dealing with ethics, and are often less experienced with regard to ethical questions.

Despite the familiarity of many researchers with questions of ethics and a broad acceptance of the need for appropriate ethical sensitivity, there is growing scepticism whether the current ethics governance infrastructure is fit for purpose. Ethics review procedures are viewed as cumbersome, bureaucratic and stifling research. This type of view is encapsulated by Klitzman (2015) who used the provocative term “ethics police.” Schrag (2010) goes further and uses the term “ethical imperialism,” albeit in the context of social sciences. This paper argues that ethics in neuro-ICT does not have to be a top-down imposition.

The scepticism concerning current ethics processes can grow exponentially in cases where precedents are not clearly established because the research is breaking novel ground. In such cases, it may not be clear what exactly constitutes an ethical issue, how it could be identified, and what actions should be taken to address it. This type of situation can arise when research is undertaken across and between disciplines, especially where these disciplines have different views of the nature of ethical issues and ways of engaging with them.

In this article, we, therefore, ask whether there is a way of conceptualizing and addressing ethics that is sensitive to the often complex issues that interdisciplinary or transdisciplinary research in neuro-ICT can raise without falling prey to the ethical imposition of which current research ethics processes are sometimes accused. We propose a conceptualization of ethics based on discourse ethics, and demonstrate that this allows for a broader view encompassing established ethics procedures but remaining open to additional influences. We then demonstrate the relevance of these ideas by exploring how they have been implemented in a research project to which all potential exacerbating factors apply, i.e., complexity, multidisciplinarity, and uncertainty. The project we present as a case for this approach to ethics is the Human Brain Project (HBP), a European-funded ICT Flagship with a duration of 10 years and a financial value of several 100 million Euros. We reflect on the HBP as its participants responsible for empirical research on and practice of ethics. Our position within the project provides us extensive and deep knowledge of the HBP, while at the same time requires to remain reflexive regarding practical implementation challenges and ways of addressing them.

We argue that the HBP shows that a dialogical conception of ethics is not only possible but may well be the only practical way of dealing appropriately with ethical issues in modern large-scale technology-enabled projects. We suggest that this example may help to develop good practice in future approaches to such projects. The contribution of the article is thus partly theoretical in that it proposes a novel way of understanding ethics in neuroscience research, but it is also practical in that it explores innovative approaches to tackling these issues.

To make this argument, we begin with a review of the various bodies of literature with a bearing on the ethics of research undertaken in large-scale data-driven projects focused on neuroscience. We then discuss the dominant approach to dealing with ethics on a project level, namely the institutional review processes and their weaknesses. On this basis, we then introduce the idea of an open and discourse-based approach to ethics. We then explain how these ideas can be implemented using the example of the HBP. We specifically focus on the question of how the project engages and aligns various overlapping discourses, with a view to finding appropriate solutions. This, we argue, constitutes the dialogical approach to ethics that modern and highly complex data-driven research in neurosciences requires. We conclude the article by discussing limitations and further developments of the approach.

## The Ethics of Neuro-ICT

Current neuroscience research produces large amounts of data. Much neuroscience research aims to leverage this by applying what can be termed “big data” approaches, i.e., using data analytics tools and methods to gain new insights. The immense complexity of the brain requires such approaches to develop novel insights. Such “big data neuroscience” work draws on a number of scientific fields with distinct traditions and cultures including neuroscience itself, medicine and computer science.

We use the term neuro-ICT to denote this type of research: approaches that depend on neuroscience as well as computer science to collect and analyze data. The proliferation of new large-scale brain research initiatives indicates the trend towards neuro-ICT. It is driven by new technologies and neuroscience tools which allow for the collection of hitherto unknown quantities of data which in turn require novel approaches for visualization and analysis. Concomitantly, one can observe a growing interest in neuro-ICT from the field of computer science where such work is seen to hold the promise of new computing processes and paradigms that overcome the limitations of current technologies, e.g., in the field of artificial intelligence. The term neuro-ICT thus includes activities that are specifically geared towards the use of ICT in neuroscience, such as neuroinformatics, but also ICT-oriented neuroscience, such as big data approaches to neuroscience and neuroscience-oriented ICT research, such as the continuing development of brain-computer interfaces and work in neuromorphic computing.

There have been intensive discussions about ethics within each of these reference disciplines of neuro-ICT. Ethical discussions can also be informed by other input, such as the professional codes of ethics (e.g., the ACM Code of Ethics), the social and institutional position of researchers or wider public debates. Understanding the ethics of neuro-ICT research requires an awareness of the ethical discourses and traditions in each of these fields. This section, therefore, starts by highlighting some of the key approaches and topics in each of these areas.

### Sources of Ethical Insights

The most prominent source of ethics in neuro-ICT is probably the discourse on biomedical ethics. Going back at least to the time of Hippocrates, biomedical ethics has produced a large body of work to inform both biomedical practice and research. During the 20th century, this was formalized in the Nuremberg Code (Freyhofer, 2004) and subsequently in the Helsinki Declaration (World Medical Association, 2008). A very influential milestone was the Belmont Report (The National Commission for the Protection of Human Subjects of Biomedical and Behavioral Research, 1979) which provides the basis for much of the practical implementation of biomedical ethics. While there are many different views on biomedical ethics, it is probably fair to say that it is currently informed by a mid-level theoretical approach sometimes called principalism (Clouser and Gert, 1990), which is based on the assumption of a universally shared common morality which allows the identification of principles, rules and obligations that can guide practical decisions (Beauchamp and Childress, 2009). Key principles are beneficence, non-malficence, autonomy and justice.

This brief overview cannot do justice to biomedical ethics and the enormous body of work that underpins and defines it. It is important to recognize that biomedical ethics arose from a more general discussion of ethics understood as the discipline of moral philosophy. Moral philosophy is probably as old as humanity and deals with the question of what is right or wrong, what we should or should not do. In current discussions one can often see references to some main streams of ethical thinking, notably to consequentialism or utilitarianism (the theory that holds that the moral status of an action is determined by its consequences; Bentham, 1789; Mill, 1861; Verbeek, 2001), deontology (the theory that sees adherence to duty as the main characteristic of ethics; Kant, 1788, 1797; Fins, 2009) or virtue ethics (which sees the character of the agent as the main determinant of ethics; Foot, 1978; Aristotle and Barnes, 2004; MacIntyre, 2007). In addition to these main streams, there are numerous other theoretical perspectives on ethics, including some developments of these such as discourse ethics, which will be discussed below. Biomedical ethics has developed in conversation with these ethical theories and integrates many of their insights, as Beauchamp and Childress (2009) demonstrate.

In addition to the rich history and intellectual roots of biomedical ethics, there is a large body of work that has sought to formalize and implement biomedical ethics. Based on the work undertaken by the World Medical Association, as described above, there have been many efforts to implement biomedical ethics and turn these into established rules and processes. The Council of Europe (1997), for example, passed the Oviedo Convention aimed at protecting human rights in the biomedical field. Most countries with a well-developed biomedical research sector now have ethics-related processes that govern such research and that are based on these principles outlined above. However, biomedical ethics is not the only specialized field of applied ethics with a bearing on neuro-ICT research.

Neuroethics, a relatively new field of inquiry or sub-discipline (Marcus and Dana Foundation, 2002; Leefmann et al., 2016), can best be understood as an offshoot of biomedical ethics which focuses in particular on ethical issues related to the brain. It is sometimes described as consisting of two main strands: the ethics of neuroscience and the neuroscience of ethics (Roskies, 2005, p. 18). A more recent definition adds a third component of neuroethics (Greely et al., 2016, p. 637): “the neuroscience of ethics; the ethics of neuroscience research; and, most frequently, the ethical, legal, and social implications of advances in neuroscience.” One of the reasons for the development of neuroethics is the nature of the brain which is typically seen as the seat of human identity and consciousness. The moral value of identity and consciousness warrants the specific attention of a sub-discipline of ethics. (Ackerman, 2006). Neuroethics covers a broad range of issues, all of which gain additional relevance or display different angles because of their link with the brain. These range from fundamental questions such as freedom and responsibility (Churchland, 2005) or the enhancement of human capabilities (Farah, 2005) to specific issues related to particular interventions, such as deep brain stimulation or novel privacy concerns related to new types and quantities of data.

Bioethics and neuroethics are closely interrelated and interdependent. They are not the exclusive basis of normative reasoning in neuro-ICT. The ICT side of this term can also refer to a long history of ethical deliberation, typically referred to as computer ethics, cyberethics, information ethics and similar terms (Bynum, 2010). The issues discussed in these discourses overlap to some degree with those in bioethics and neuroethics but they also differ in various ways. The discussion of computer ethics can be traced back to the very beginning of digital computing (Wiener, 1954, 1964). However, it has gained more prominence with the more wide spread adoption of computers and the spread of the internet since the 1990’s. A recent review of the computer ethics literature (Stahl et al., 2016) showed that there is a set of relatively stable concerns that have been discussed over the last decade or so. The most prominent one of these is that of privacy, followed by professionalism and work-related issues, questions of autonomy, agency and trust. There is some discussion of specific computing technologies and the issues they raise but also of identity, inclusion, digital divides, security, harm, misuse, deception, health-related issues and justice.

This brief introduction into key foundations of normative insights in neuro-ICT aims to demonstrate the diversity of ethical positions, topics and theories that influenced the field, but cannot provide a comprehensive overview. There are other sources of normativity, such as professional ethics or general philosophical ethics that can provide further input into the understanding of ethics in neuro-ICT. The overview is nevertheless sufficient to provide the starting point for understanding the current state of practical ethics methodologies, which is discussed in the next section.

### Current Research Ethics: The IRB/REC Approach

The practice of dealing with ethical questions in research is increasingly standardized across disciplines and national and cultural boundaries. We use the term “research ethics” to denote this standard approach. Research ethics, as we understand the term, is based on the philosophical reflections indicated in the decades of discussion of biomedical ethics. It is important to understand, however, that it has been influenced in substantial, culturally and historically-contingent ways. This is most notably the case in the US American context where research ethics developed as a way to retain scientific independence and avoid stronger research regulation by the state. Its conceptual basis remains the Belmont Report (The National Commission for the Protection of Human Subjects of Biomedical and Behavioral Research, 1979) and its principles and practices, which have emerged in large part from the federally funded US research environment.

Research ethics as we understand it here is one way of putting theoretical ethical reflections into practice. It can, therefore, be seen as an example of applied ethics, similar to computer ethics or neuroethics. Unlike these other fields of applied ethics, research ethics is highly formalized and institutionalized. As we are about to argue, research ethics has moved very close to compliance, which is one of the reasons it is perceived as an imposition. It is important, however, to recognize that as a field of applied ethics, research ethics is strongly anchored in philosophical ethics and arises to a large degree from biomedical ethics. This raises the question of why an approach to applied ethics can turn into something that is perceived as problematic and how this can be reversed or avoided in different fields. Before we can respond to these questions, we need to explain the current realization of research ethics in further detail.

The practice of research ethics that most active researchers in the area of neuroscience will be familiar with is that of external review by an independent body. This body tends to be called an institutional review board (IRB) in the US, or a research ethics committee (REC) in many European countries. The operational principles remain similar, however. The leader of a research project that may raise ethical issues or receives certain types of funding is required to submit a statement explaining the ethical issues of the research, how these are to be addressed, and requesting approval to undertake the study. This application is then reviewed by the IRB which may approve, reject or require further development in preparation for a repeated review. Once the ethical issues are appropriately described and addressed, the researcher is given the approval to proceed. When unforeseen ethical issues arise, the researcher is required to raise these with the IRB. In some cases, a regular update of the procedure is required, especially for long-term studies. This approach to research ethics originates from biomedical research and is increasingly used across disciplines. The Menlo Report (Bailey et al., 2011) is a high profile example of the attempt to broaden the biomedical research tradition to cover ICT research as well.

The current research ethics regime has numerous advantages. It is relatively clearly defined, allows researchers to deal with the issues they are likely to face, limits liability and thereby provides the basis for a broad range of research activities. Over the last two or three decades, it has developed into a veritable industry with stratified decision-making bodies, from national ethics committees which tend to look at the principal questions and institutional boards which make practical and operational decisions. It has spawned specialist journals, qualifications and degrees and led to the establishment of professional identity and a body of knowledge held by those who are specialists in the field.

As this approach to dealing with ethics in research has broadened, first to cover the entirety of biomedical research, and secondly to go beyond the disciplinary boundaries for which it was devised, it has also encountered severe criticism both from within and outside the biomedical field. The most stringent recent criticism of the IRB resume from within the biomedical field was likely that formulated by Klitzman (2015). He argues that it is not clear whether the additional administrative burden that research ethics review is put upon researchers actually fulfills the main aim of research ethics, which is the protection of the research subject, the human being or non-human animal that is used to supply the data. The administrative burden and costs associated with the IRB review process and not just a nuisance, but they actually constitute an ethical issue themselves. Resources spent on ethics cannot be spent on the actual science. Where science offers the hope of ethically valuable insights, such an impediment to science requires an ethical justification (Poline et al., 2012). In attempting to address the issue of the administrative burden, the ethical review process may be outsourced to external companies, which not only removes consideration for ethics from the scientific process but may also unnecessarily incentivize approvals (Musoba et al., 2014).

Furthermore, research ethics through IRB review has both conceptual and empirical shortcomings which must be considered. One conceptual issue is that of the limitations of knowledge. IRB decisions are typically made on the basis of prior knowledge of the members of the IRB panel. This knowledge is partial by necessity, and may not cover relevant aspects of the study in question. Moreover, some possible consequences may be simply unknown and therefore outside the scope of the IRB review. This problem of missing knowledge is linked to the principle of peer review, upon which IRBs are based and which may not provide the required input and insights to best deal with the relevant issues.

There are numerous practical concerns with regard to the current research ethics processes. The reliance on* a priori* applications can lead to a tick box mentality which opposes the idea of ethics as a form of reflective practice. By separating ethics from the research process, there is a danger that it is perceived as a specialist activity which can be outsourced and divorced from the scientific research itself. The structure of decentralized ethics reviews means that there may be an inconsistency between different IRBs as well as between IRBs in different jurisdictions, leading to the potential for “jurisdiction hopping” to avoid or circumvent particular regulations (Stark, 2011). The focus on the protection of the individual participant can lead to the obfuscation of bigger ethical issues, which may be more pressing but which are rendered invisible by the process (Landeweerd et al., 2015). This, to some degree, can immunize research from critical scrutiny.

The extension of the biomedical process of research ethics beyond that field of practice may be most problematic. Schrag (2010) offers a strong critique of the expansion of the IRB model to the social sciences. Existing IRB processes not only fail to understand the specifics of social science but more importantly, it is unclear whether the underlying assumptions of research ethics are applicable to social sciences. The protection of the individual research subject, for example, an uncontroversial aim of biomedical research ethics, may not be appropriate in the social sciences. A social scientist may collect data from a respondent specifically to expose their activities, which may be detrimental to the research subject but desirable as a legitimate aim of social sciences. Similarly, it is less than clear whether the core principle of individual informed consent that guides research ethics is an appropriate way to deal with ethical questions in ICT. In the age of “big data” and automated processing, it is an open question whether the balance between the benefits and detriments of research can best be struck by asking individuals about the use of their data, which they are unlikely to understand in detail, and which may be subject to change. Another problem is that research ethics is based on the assumption that the aim of the research is essentially desirable and ethically valuable. The focus of research ethics is therefore on the process of research, and not its outcome. In biomedical research where the aim is typically to understand diseases to help heal them, this premise may hold. However, even in biomedical research, it is often less than obvious that the outcomes of research are desirable. In other areas, including ICT research, the premise that the intended or possible outcomes of research are of ethical value may be incorrect and an ethical approach to research would need to incorporate reflection on the products and outcomes as well as the process of undertaking the research.

We have aimed to demonstrate that the current and dominant approach to ethics in research is open to a number of important and legitimate questions. At the same time, it is increasingly institutionalized and becoming further entrenched across all academic disciplines. The IRB process of research ethics that was meant to balance the power of researchers, as well as powerful institutions such as pharmaceutical companies, is now becoming exceedingly powerful itself, colonizing the research process and creating new ethical issues. The nature and content of the concepts and ideologies being extended in this fashion relate directly to the dominant power, which is in this instance at least partly represented by biomedical ethics processes and the historical trajectory of scientific research in a U.S. context. Having described the shortcomings and ethical concerns of the research ethics process, we now proceed to a discussion of potential alternatives.

### Beyond the IRB: Discursive Approaches to the Ethics of Research

The key problem of current research ethics is that to some degree it achieves the opposite of what ethics should do. Instead of opening up, questioning and debating ethical questions, it closes them down and removes them from critical scrutiny. Furthermore, it removes reflection upon ethical issues from the research process and makes shared forms of responsibility impossible. These issues are problematic in any area of science and research. They are even more problematic in novel and emerging areas such as neuro-ICT where different disciplines and intellectual traditions come together, when novel problems and questions may form and where established answers may not suffice.

To move beyond the IRB approach, we believe that ethical methodologies will need to be open, inclusive and discursive. These principles are of course not new, and we can build on several theoretical positions and discourses to justify and develop them (Ess and Thorseth, 2006). We will briefly outline two of these to indicate our theoretical position: one is Habermasian discourse ethics, and the other is the current debate around Responsible Research and Innovation (RRI). We focus on RRI as an approach promoted by the European Commission that has informed the structure of the work described later. Without being able to make this argument here in detail, we submit that discourse ethics can be seen as a key theoretical root of RRI.

Discourse ethics constitutes an ethical theory that is conducive to our interest in overcoming the perceived limitations of the IRB tradition. Discourse ethics is closely linked to the work of Apel (1990, 2002) and Habermas (1991, 2006). It is based on Kantian deontology, i.e., on the assumption that the principles of ethics are located in the agent’s intention to do the right thing, to do their duty. Kant’s (1788, 1998) version of this principle is embodied in the Categorical Imperative, which holds that one should always act according to the maxim that one could wish to be the basis for general legislation (Myskja, 2008). Discourse ethics recognizes that it is beyond the ability of any individual to truly achieve this and therefore moves the basis of ethical deliberation away from the individual and towards the group of individuals who are potentially affected by an action. As a consequence, Habermas holds that only those norms can claim to be valid that meet (or could meet) with the approval of all affected in their capacity as participants in a practical discourse.

This article does not offer the space to discuss these ideas in the depth they deserve. Suffice it to say that discourse ethics offers a theoretically sound starting point for moving from a traditional and individual-centered view of ethics to one where agency and responsibility are distributed across various stakeholders. Discourse ethics has been widely discussed and there are relevant examples of its application to ICT (Stahl, 2008; Mingers and Walsham, 2010; Rehg, 2015). It is also worth noting that discourse ethics is closely linked to Habermas (1981) theory of communicative action, which is an important theoretical contribution to critical theory which, in turn, has inspired a recent discussion concerning critical neuroscience (Choudhury and Slaby, 2011; Fitzgerald et al., 2014; Schleim, 2014).

A second approach that informs our attempts to move beyond the IRB-centric tradition of ethics is that of the currently widely-used concept of RRI. This can be considered as a contribution to the discussion of research and innovation governance which is of particular importance to large research funders. Increasing investments in research and innovation raise the question of whether these investments are palatable to the citizens and tax payers who have to pay for them. Public opposition to some types of research, such as nuclear power, genetically modified organisms (Dąbrowska, 2007), or geoengineering (Macnaghten and Owen, 2011), have caused funders to consider the question how public acceptance of research, innovation and their consequences can be assured. The concept of RRI, which originates from several research funding organizations (e.g., the European Commission) as well as multiple scholarly disciplines such as science and technology studies, technology assessment and philosophy is an attempt to provide an answer to this.

RRI has been defined as “the on-going process of aligning research and innovation to the values, needs and expectations of society” (Rome Declaration, 2014). There are different scientific and policy interpretations of what exactly this means. The European Commission is promoting an interpretation of RRI that focuses on six different keys or policy areas: gender equality, ethics, science education, open access, public engagement and research governance (European Commission, 2012, 2013). The UK Engineering And Physical Sciences Research Council (EPSRC) has established a framework for RRI that is encapsulated in an acronym called AREA, which stands for anticipation, reflection, engagement and action (Owen, 2014). This, in turn, is based on the work by Stilgoe et al. (2013) which underlines the collective responsibilities that various stakeholders assume in the research and innovation processes, and that require a strong emphasis on responsiveness and the ability and willingness to engage with other views and voices. The AREA framework is implemented in the HBP.

Elsewhere we have suggested that RRI may best be understood as a higher level responsibility or a form of meta-responsibility (Stahl, 2013). This means that RRI takes as its point of departure the existing multitude of established relationships of responsibility. All agents in research and innovation environments are already entangled in a variety of responsibilities, including the researcher’s responsibility for the appropriate collection and analysis of data and research conduct, the research institution’s responsibility for transparent expenditure of research funding, or the funding organization’s responsibility for allocating resources according to appropriate criteria. RRI embodies a view of this existing ecosystem of interlinking responsibilities that is used to shape, maintain, develop, coordinate and align these existing responsibilities with a view to ensuring that the processes, products and purpose of research and innovation are acceptable, desirable and sustainable.

The reason for this brief introduction to discourse ethics and RRI, which cannot do justice to either of these complex notions, is to demonstrate that there are approaches to ethics and to dealing with possible risks and downsides of research and innovation which can look very different from the established research ethics process. They move away from a linear and expert-centered view of ethics towards a more open, discursive and responsive approach. In these communication-oriented views of ethics, it is not necessarily assumed that ethical issues can be identified in advance. It is also not taken for granted that the objective of the activity is beneficial and acceptable to the relevant stakeholders. Instead, an open exchange between researchers and stakeholders is employed to identify and highlight possible issues. The responsibility for addressing these issues is not taken for granted, but can also be subject to the discussion.

These ideas of an open, discursive approach to ethical issues underpin our approach to ethics in large neuro-ICT projects which we now describe using the example of the HBP.

## Dialogues About Ethics in the HBP

The HBP is a large European research project that is funded under the Future and Emerging Technologies flagship initiative. It began in 2013, and has an expected duration of 10 years. Its core funding of more than 400 million Euros sets it apart as one of the larger international initiatives in this field. Its three main areas of interest are neuroscience, medicine and ICT. The initial emphasis of the project on a bottom-up brain simulation platform has given way to the broader aim of developing an ICT infrastructure for neuroscientific research. In the early phases of the project, there was some high-level debate about its aims and their justification (Frégnac and Laurent, 2014), which seems to have subsided, leaving the project to focus on research and infrastructure building (The Lancet Neurology, 2017). The project is organized into 12 subprojects, four of which focus on neuroscience, six on the development of ICT infrastructures, one on management and administration, and one on matters of society and ethics (Amunts et al., 2016).

The authors of this article are all members of this final subproject, which is divided into four work packages. The Ethics and Society subproject is tasked with implementing RRI throughout the HBP. For this purpose, it includes work packages focusing on anticipation and foresight, philosophical and new ethical reflection, public engagement and the management and support of project members with regard to ethical issues. These different components interact closely, but for the purposes of this article, we focus on the final one, Ethics Support. The Ethics Support function emerged after the initial phase of the project in response to a call for ethics management that was aimed to address shortcomings in compliance. It quickly became apparent, however, that a top-down imposition of compliance principles could not do justice to the complexity of the project. Ethics management therefore merged into Ethics Support which is modeled on the idea of a dialogical concept of ethics, as described above. This adoption of the dialogical principle did not follow a master plan but was the result of numerous conversations of the individuals working on this task with scientists, project officers and external stakeholders. Before we discuss these in detail, we shall briefly introduce the ethical issues that were identified and are currently being addressed.

### Ethics in the HBP

From the outset, it was clear to the consortium planning the project that the HBP would require a strong emphasis on ethics. The Ethics and Society subproject was, therefore, part of the original conception of the project and receives a level of funding that is approximately 3% of the overall budget. The conceptualization of ethics that motivated this early attention to such issues was not linked to a particular ethical theory or tradition, but broadly construed and based on the awareness that the HBP had the potential to raise ethical concerns. These ethical concerns were certain to include traditional research ethics questions, but they were clearly broader than research ethics.

The types of ethical issues that the project encounters range from the well-described and understood (e.g., human subject protection) all the way to more speculative questions around the possibility of a brain simulation achieving the status of personhood (Lim, 2014). A first overview of the various issues that are specific to the type of work undertaken by the HBP was offered by Christen et al. (2016). Evers (2017) offers an account of the contribution of neuroethics to this type of work, whereas Aicardi et al. (2018b) discussed the broader questions of how ethical concerns can be reflected appropriately in this type of research. A general overview of the approach to ethics in the project is provided by Salles et al. (2019).

To give a quick overview, one can distinguish between the various types of ethical issues that the HBP encounters. These include traditional research ethics questions, notably the protection of human research subject and of non-human animals. This category also includes research on human cells and research in non-EU countries. Ethical issues more closely related to the functional programming side of the project include compliance with the new EU General Data Protection Regulation. This is a comprehensive reform of data protection rules that seek to harmonize the processing and regulation of personal data in the EU single market (European Parliament, 2016). To some extent, scientific research is given a “privileged” position under the GDPR with the application of several derogations. It is therefore important that new and enhanced provisions are adhered to as a model of legal and ethical compliance, and to serve as a beacon of good practice. The HBP can potentially give rise to questions of research integrity and data confidentiality that can be seen as part of a larger set of questions revolving around the governance of data. Related to this are questions of intellectual property. Broader social concerns arising from the work undertaken in the HBP include the question of unintended misuse, the impact of novel technologies and insights on social processes including the role of doctors and their impact on employment. Relationships with various communities involved in the HBP are important, not least concerning the development of the HBP infrastructure. Finally, it is worth mentioning there are fundamental philosophical questions such as the nature of consciousness which have important clinical implications, but which are also of ethical relevance in that they inform our individual and collective understanding of our identities as human beings.

Most of these ethical issues are not particularly surprising or novel. What sets them apart and makes them an interesting topic of investigation is their intersection and overlap. Some of them are clearly linked to particular research traditions, but in the HBP they are faced by a highly diverse audience of scholars from different disciplines and backgrounds with many ways of interpreting these issues and of dealing with ethics in general due to the varying research environments in which scientists are socialized. A further interesting aspect of these ethical issues is that they are partly located in what we referred to earlier as research ethics, i.e., the tradition of formalized biomedical research ethics that is governed by IRBs and aims at compliance. This is the case, for example in animal research which needs to comply with animal protection regulation or human subject research, the core field of research ethics. However, even in those cases where the HBP ethical issues are clearly compliance-oriented, it is not always clear how compliance is to be achieved and demonstrated. Compliance with the GDPR is a case in point, where the question of how to apply a significant and novel piece of legislation is still subject to societal debate. Similarly, questions of dual use, i.e., the use of neurotechnologies for military, security or related applications form part of the EU’s research ethics framework, but, as the Society and Ethics group found out, this does not imply that it is clear how to deal with such issues.

One important factor with a significant influence on how ethics is addressed within the project is the ethical framework of the European Commission. As an EU-funded project, the HBP is subject to the principles of research governance laid out by its funder. The EU has instituted a system of ethical review that is based on the biomedical model. Its starting point is an ethical screening of all successful proposals. Where any ethical issues are identified, this can lead to audits and the definition of the additional deliverables that describe how they are addressed. The HBP as a large-scale project that raises the numerous issues listed above, and is subject to stringent and frequent ethics checks that run alongside the scientific and technical reviews. To a large degree, the HBP is therefore subject to the traditional top-down ethical approach described earlier. However, our experience shows that this approach is not sufficient. For the practitioners in the project to understand and appropriately deal with ethical issues, the dialogical approach advocated in this article is required, as we will demonstrate in the following section.

### Ethical Dialogues in the HBP

The dialogical approach to ethics that we have introduced requires a practical engagement of the various stakeholders in an open and reflective way. Instituting such a dialogue or series of dialogues requires the identification of all stakeholders. It is important to understand that such a dialogue does not take place in a vacuum, but is located in a specific historical and social context. Understanding this context requires an awareness of both the relevant literature and current public debates. The Ethics and Society sub-project of the HBP pays specific attention to future-oriented accounts as part of the work of the Foresight Lab (Aicardi et al., 2018a) as well as the philosophical and neuroethical debates (Evers, 2017). A further central role is played by the public engagement activities that reach out to specific stakeholder groups and society at large. These can be interpreted as part of the broader discourse concerning ethics in the project.

In this section, we focus on the various intersecting and overlapping dialogues within the HBP that form part of the HBP’s ethical activities. We describe the principles of discourse, key stakeholders as well as some processes and outcomes. It is not possible here to comprehensively describe all ethics-relevant discourses in a project of the size and complexity of the HBP. In addition, we describe these discourses from the point of view of the Ethics Support group. This should not be read to imply that this is the exclusive core of all ethics in the project, but is a simple methodological choice determined by the fact that the authors of this article have the closest insight into these discourses. We concede that other descriptions of the HBP ethics discourses are possible. By positioning the Ethics Support group at the heart of the HBP ethics discourses, we can describe it as an interface that facilitates the cross-fertilization of several of the other discourses. Ethics Support, as indicated earlier, is one of the four work packages of the ethics and society sub-project. Its purpose is to ensure that ethical issues are dealt with to the highest standards. In order to facilitate this, Ethics Support needs to be aware of current issues. The point that we want to introduce here is that Ethics Support interpreted as an implementation of research ethics and focused on compliance does not do the ethical complexity of a project like the HBP justice. While Ethics Support is the home of research ethics in the HBP, and compliance forms part of its activities, even compliance in the most narrow sense requires a broader understanding of ethical issues in a quickly changing external environment. In addition to Ethics Support’s own research on the current state of the art with regard to various ethical issues, the group can benefit from the insights generated by the neuroethics and philosophy group who are experts in the current debate in this field (Salles et al., 2018). Similarly, insights about likely and possible futures can be gleaned from the Foresight Lab. In theoretical terms, this means that Ethics Support needs to be cognizant of biomedical and research ethics, but it needs to move beyond these to understand current societal discussions. As a result, an ethics of dialogue is a more suitable theoretical underpinning than a narrow view of research ethics. Only such a focus on dialogue will allow gaining the insights required to interpret issues appropriately and deal with them. Work undertaken within the HBP is one source of such insights.

A further important source of ethical insights which is crucial to the discursive construction of the ethical position of the HBP is a set of discourses with external stakeholders. These are undertaken by the Public Engagement group of the HBP, which reaches out to specialist audiences, such as relevant scientific and technical communities, but also to potential beneficiaries of the work, e.g., patients and their representatives as well as civil society at large. These engagement exercises provide important insight into concerns and priorities of external stakeholders who should benefit from the work undertaken in the HBP and the values that these external stakeholders hold.

In order to comprehend current concerns as well as practices of dealing with ethics, Ethics Support has instituted a number of specific means of communication. One of these forms part of the ethics compliance processes. To understand the state of compliance-relevant activities, i.e., those activities that are clearly regulated and legislated, all principal investigators (PIs), i.e., all task leaders of the HBP are sent an online survey to ask them about the state of compliance-related activities. This discourse is highly structured and aligned with the EC’s H2020 ethics self-assessment process (European Commission, 2014). While this conversation starts out in a very structured way, it then often develops into a more wide-ranging discussion where PIs raise queries, Ethics Support comments on documentation, asks for clarification etc. until a shared understanding of the various activities is achieved.

These compliance processes form an important aspect of the discourse with the EC about the received orthodoxy in terms of addressing ethics. This discourse takes place through a number of ethics assessments, reviews and audits that take place at predetermined stages of the project, sometimes two or three times per year. The EU GDPR mentioned earlier provides for a techno-legal approach through early implementation of sustainable privacy-enhancing technologies that enable legal compliance throughout the life cycle of data. This primarily involves conducting privacy impact assessments at the start of each research in order to assess the risks and severity of data processing on the rights and freedom of natural persons. Following this is establishment of privacy by design and privacy by default procedures through the development of adequate technical and organizational measures such as pseudonymization and data minimization designed to implement data protection principles and protect the rights of data subjects in a manner that allows for legal compliance in a modern world (European Parliament, 2016).

While the term “compliance” seems to suggest a rigorous and predetermined view of ethics as described earlier as the traditional research ethics approach, the practice of this discourse is fundamentally more open. In many cases, it is not clear which rules apply, who can determine the application or how they need to be implemented. This is partly caused by the international and cross-jurisdictional nature of the HBP and partly by the rapidly moving scientific and technical development which can render precedent difficult to interpret. The compliance discourse furthermore takes place with ethics reviewers, who are external experts contracted by the EC to assess the HBP’s work. These external experts have different areas of expertise and interests, and change over time, leading to a level of fluctuation in terms of topics and positions.

Despite this, the compliance-related discourse remains relatively fixed and externally determined in terms of structure and content. Ethics Support, therefore, employs several additional discourses. One of these focuses on the external expertise offered by the Project’s Ethics Advisory Board (EAB). Members of the EAB are independent experts whose work receives administrative support. Expertise in the EAB covers many of the key ethical issues including animal research, human subject protection and data protection. As the project developed and novel issues became evident, membership of the EAB has changed to reflect this, leading to the inclusion of experts on the ethics of robotics and information systems.

The discourses described so far involve external members, but there are additional discourses focusing on HBP-funded scholars as well. For example, as part of its own research activities, members of the Ethics Support team interviewed the leaders of all subprojects in order to understand their views of ethics. Subsequent to this, we surveyed all project members using an online survey as a way of accessing their views, questions and priorities. This discourse highlighted the issues that perceptions of ethics differ between disciplines and subprojects, and that a more continuous engagement with the scientists in the project was required. As a consequence, and following suggestions from the EAB, a group of individuals was assembled to represent all of the HBP’s subprojects, and whose role was to serve as partners of Ethics Support in all ethics-related issues. This group, the so-called Ethics Rapporteurs, provides the subject expertise and detailed knowledge of project operations, they can communicate with their peers in their subprojects about ethics and contribute their understanding to the discussion.

Each of the groups mentioned above has internal discourses around ethics, which involve the Ethics Support team as a common factor. In addition, we put other measures in place to ensure that the discourses cross-fertilize each other. For example, the EAB members are paired up with the Ethics Rapporteurs and continuously discuss and maintain an awareness of the current state of ethical issues in all subprojects. EAB members and Ethics Rapporteurs participate in the regular ethics reviews, and thereby interact with the EC and its ethics reviewers. The EAB feeds into the discussion of the Ethics Support group and the Ethics and Society subproject as a whole, informing agenda-setting decisions and providing feedback on research and recommendations. Another important example of cross-fertilization is Researcher Awareness activities that are a shared responsibility between Ethics Support and Foresight to facilitate reflections on potential ethical and social implications of research within HBP.

**Figure 1:**
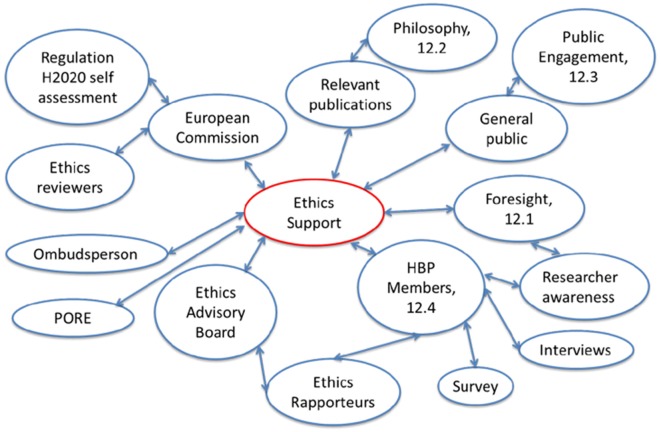
Key participants and communication links of the Human Brain Projects (HBPs) ethics discourses.

Figure 1 gives a high-level overview of the various groups engaged in the discourse and highlights key communication links.

This figure is not necessarily complete, as there are numerous developing discourses between the participants listed here and others which may not be listed. We realize that it is only one possible way of representing the discourses in the HBP, and that there will be many others. We have put Ethics Support in the center of the diagram because it is from this perspective that we have written the article, but are clear that this does not imply that all discourses revolve around Ethics Support or that Ethics Support “owns” ethics in the HBP. The purpose of the diagram is only to highlight some of the main discourse participants and the range of stakeholders both within and outside the HBP that provide input into ethics discourses.

## Critical Reflections and Discussion

The previous section described the ethics-related discourses in the HBP and the fundamental role of RRI in the creation of the Ethics Support group’s approach to ethics in the Project from an overarching, generalized perspective by necessity. We outlined how our approach overcomes some of the limitations and ethical issues associated with the traditional IRB orientation. This discursive framework is not without limitations or potential pitfalls, and we discuss some of these issues and their implications in the next section.

### Limitations and Problems

Numerous challenges are present with regard to the application of the approach which we explore here. One immediate, practical concern is that of cost and resource implications. Creating and maintaining an embedded Ethics and Society subproject with an integrated Ethics Support group is considerably more expensive than engaging in routine, pre-project box-ticking, to say nothing of the administrative burden of multiple yearly ethics reviews and other aspects of ethics management.

The resource requirements are not only a constraint that may make it difficult for other projects to institute similar approaches, but they can lead to frictions and consequently to doubts about the legitimacy of engaging with ethics in the first place. Resources spent on instituting ethics-related discourses are resources that cannot be spent on science. In a traditional view of science that sees any scientific endeavor as automatically ethically justified due to its positive impact on knowledge production, doubts can arise concerning the necessity of engaging with ethics. This issue is highly dependent on the personal and disciplinary background of the researcher, which also play a role in the next point.

An overarching concern is that of awareness; for example, members of the HBP possess varying levels of awareness both of ethical issues and the processes required to address them within the project context. This is linked to other factors which we have already mentioned, including the differing socialization of researchers in different disciplines, the complexity of the project as a whole, and the variability of Member State requirements for research. Through the established communication channels and overlapping relationships between external parties and internal ethics governance frameworks, we must continuously engage in clarification and modification of the processes and attempt to raise awareness of these. The willingness of researchers to engage in discourse around ethics and innovation is also a considerable potential barrier. Researchers face many pressures on their time, and a prolonged exchange about the ethical issues presented by their research may not be a high priority. Furthermore, a view of research ethics as a barrier to progress is pervasive amongst some groups, and that presents an additional challenge.

There are also questions of authority, with regard to ethics and for the scientific research process and outcomes. Both may be addressed to some extent through a discursive negotiation and reinforcement of shared responsibility, but the likelihood of success of extended dialogue is highly contextual. Somewhat related to this are issues of conflict, how aspects of the ethical process should be enforced if clashes occur, and what sanctions may be necessary should a serious issue arise.

As a way of mediating the impacts of these issues, the HBP Ethics and Society programme chose to highlight the practical relevance of RRI to HBP researchers, and to raise awareness of this through our external and internal outreach and community-building activities. We emphasize the benefits of more sustainable, socially-relevant research that resonates with multiple stakeholders. However, institutionalization of ethics dialogues (e.g., compliance) and RRI can also lead to bureaucratization and standardization and, thus, encounter similar criticisms as those raised in the debates on “ethics police” and “ethical imperialism.” Part of demonstrating this relevance is our key role in supporting the production of legally and ethically-compliant research. This is partly because enforceable issues are simplest to justify in a complex, multidisciplinary project involving multiple countries, but also because the HBP is publicly funded and maintaining public trust and the societal “license to operate” is important. However, as we have hope to have shown, the Ethics Support work and the programme of RRI of which it forms a part are much wider than legal compliance.

In the same vein, another challenge is presented by the nature and legal context of the project’s funder, the European Commission. Ethical processes and governance in the HBP are, by necessity, shaped by European and international law, which can be interpreted as ethical impositions (Anghie, 2007). It is clearly beyond the scope of this article to provide a critique of international and European law and the regulatory framework of European research. Suffice it to say that from the perspective of Ethics Support, they provide boundary conditions that can be difficult to negotiate.

The inability to fully escape from existing power relationships could also be used to criticize our overall approach. We draw on the concept of RRI, and have argued that it can be justified with reference to Habermasian discourse ethics. However, we are aware that there is significant criticism of discourse ethics, not least because of its reliance on the ideal speech situation. This is the counterfactual scenario where all participants in a discourse have the opportunity to share their views and modify their positions with a view to reaching a consensus on the issue in question. Discourse ethics is clear that this ideal speech situation is counterfactual and, as Habermas puts it, it is transcendental in a Kantian sense, which means that imagining it is a necessary condition of the possibility of real discourses. To put it differently, if we could not imagine an ideal discourse, then there would be no point in a real discourse, as we would not know how to structure it. This leads us into deep philosophical territory which goes beyond the confines of this article. However, it is clear that the real discourses within the HBP suffer significantly from imbalances of power, given that they include numerous actors (e.g., junior and senior academics, administrators, EC officials, ethics reviewers) that are in established power relationships with each other. A free and open discourse is therefore not only constrained by the legal and institutional context, but also by the nature of extant formal and informal relationships and the perceived identities of the agents within them.

A final fundamental concern worth mention is that the open and dialogue-oriented approach we propose here is open to intentional manipulation and political misuse. By opening the debate concerning the process and expected outcomes of scientific research to a broader audience, one can inadvertently invite voices that may not be well informed but may be well organized and aim to steer research and innovation in a particular direction. It is not difficult to envisage that certain ideological or religious views would inform opposition to specific research questions or methods which could stifle freedom of inquiry. This problem is not limited to scientific research. Open and deliberative democratic societies that invest heavily in scientific research are currently subject to increased levels of public scrutiny and questioning of established truths. The established expert-based IRB process that governs scientific research, while sometimes oppressive, at least has the advantage from the point of view of scientists that it is undertaken by scientific peers, who generally do not question the very basis of the research. We concede that this is a valid concern and one that a deliberative approach to RRI will need to take into account.

### Managing the Outcomes of Ethics Dialogues: Impact and Relevance

One way of arguing that the fundamental limitations of a discourse-oriented approach to ethics discussed above are to demonstrate that the outcomes are recognizable and more effective than those that would have been achieved relying on the traditional approach. Unfortunately, it is not straightforward to measure the impact of taking a dialogical approach to ethics in neuro-ICT, particularly outside the realm of legislative compliance. At the moment, the HBP involves 116 partners from 19 countries, each of which has their own ethics regulations and requirements.

The other fundamental methodological problem in measuring the impact of ethics dialogues is that there is no independent baseline against which we can measure. It is impossible to know how the HBP would have developed if a different regime of ethics or RRI had been in place. Despite these methodological limitations, we believe that there are good arguments for supporting the positive impact of the dialogical concept of ethics that we have employed. As a starting point, there is now a widely distributed ethics infrastructure in place in the project. This includes subject expertise in many different fields and aspects of ethics as well as a number of contact points for ethics in the various scientific and technical activities. Ethical viewpoints are institutionalized at various points within the governance structure of the project. The project not only has a satisfactory way of dealing with traditional research ethics, but continues to reach out to academic and user communities and to find ways of incorporating external insights into practice. Overall, we believe that the approach has succeeded in what it was meant to achieve, namely to overcome the overly narrow traditional approach to research ethics. Consequences of the ethical discourses within the HBP can be observed in many different areas, for example in the privacy-sensitive design of medical informatics work, in the close collaboration of neuroinformatics and ethics compliance, in the appointment of a data protection officer or in the ongoing discussion with colleagues about the challenges of data governance or the concept and practical possibilities of responsible dual use.

This is not to claim that we have resolved all ethical problems in the HBP. Many of them require continuous monitoring and reflection. The fact remains that many of our discourses are practically driven and constrained by the traditional IRB approach and legal requirements which often leave limited space for discussion. However, despite the narrow space for maneuver created by of many of these legal frameworks, the use of ethics dialogues can be particularly fruitful because it presents a pragmatic way of reconciling differences across complex disciplinary, institutional and national contexts across Europe and beyond.

## Conclusion

By using the HBP as a case study, we have shown that the conceptual framework of discourse ethics and its practical application through RRI offer a viable, responsive way of addressing the ethics of international, innovative, interdisciplinary research without reproducing IRB or REC approaches. In fact, discourse ethics may present a practical method of reconciling differences within large-scale neuro-ICT projects that involve diverse disciplines, institutions, and countries whilst operating in the complex and uncertain contexts that characterize innovative research. Due to the openness and flexibility of this approach, ethics dialogues also offer the opportunity to keep pace with the development of emergent technology in a way that legislation, individual ethics, or rigorous rules cannot. We would, therefore, suggest that a discourse-oriented approach to ethics is not only a viable alternative to the present approach, but it is the only way forward.

Research is increasingly called upon to move beyond specific problems that can be solved in the lab and engage with bigger societal challenges. The world faces numerous, significant problems that will require new thinking and novel insights to be addressed. The most visible example of these issues is summarized by the United Nations as the Sustainable Development Goals (United Nations, 2015). These and other grand challenges call for research to suggest solutions and provide evidence of the solutions’ efficacy. To address such challenges, boundary-crossing collaborations from diverse disciplines, countries, sectors and stakeholder communities are needed.

This implies that the ethical and societal issues associated with such large-scale and interdisciplinary research will grow in importance. This is true for neuro-ICT research, but also for fields beyond neuroscience and ICT. We believe that addressing the ethical challenges of such work will require a discursive approach. The HBP’s way of identifying, highlighting and engaging with its ethical issues may provide an example of interest for dealing with ethics in the future.

### Contribution

We have framed this article in terms of ethical issues in large neuro-ICT projects. At the time of writing this article, there are numerous brain research projects in preparation or being implemented. They include the US BRAIN Initiative, the Japanese Brain/MINDS project, the Canadian Open Neuroscience Platform, the China Brain project and a number of further national or regional projects. In 2017, these projects met for the first time to discuss collaboration under the auspices of the International Brain Initiative^1^. The development of these numerous, large neuro-ICT initiatives underlines the importance of thinking about ethics in an open and collaborative way. The further success of international neuroscience research will depend to a large extent on collaboration between different projects, labs and researchers. This, as we have argued elsewhere, will require the incorporation of some shared understandings of ethical issues (Stahl et al., 2018). At the same time, the regulatory and ethical issues of the different projects will differ and call for appropriate bespoke solutions. Many of the ethical issues that can be expected of such collaborations working to create novel outcomes will not fall within the remit of a traditional IRB process, and we offer an RRI-informed dialogical ethical framework as a possible alternative.

In this article, we did not aim to provide practical guidance to researchers in other neuro-ICT projects on how to address particular issues. The main point was to discuss the current ethical underpinning of research ethics and to outline its limitations. We argued that a reliance on biomedical ethics and in particular on the compliance-oriented implementation of research ethics does not do the complexity of current ethical issues justice. Drawing on principles of dialogue-oriented ethics and incorporating our experience from the HBP, we demonstrated that different interpretation of ethics is not only possible but also more suitable to address current ethical issues.

This article’s contribution to knowledge is partly theoretical in that it shows how the current discourse concerning RRI and one of its theoretical roots, namely discourses ethics, can be conceptualized in large, multidisciplinary, international, collaborative and mission-oriented research. Our practical contribution is to provide an example of how these ideas can be put into practice. Many of the aspects of this example are by necessity idiosyncratic but we believe that the principles that they are based on are capable of being generalized. We, therefore, provide a practical example of implementing a theoretically grounded practice-oriented approach to RRI. Practical lessons to be learned from our work and this article, therefore, refer to the structure and implementation of ethics-related processes in large projects. We believe that the interlinked network of ethics discourses represented in Figure 1 is a suitable model for dealing with ethics. This is not to suggest that it is the best or only model, but that it contains a number of components that are worth considering when setting up ethics-related structures.

### Further Research

There are promising lines of research related to the ideas we present here. As noted earlier, the relationship between discourse ethics, critical theory as informed by communicative action (Habermas, 1981), and emergent critical neuroscience discussions (Choudhury and Slaby, 2011) would be one interesting avenue of future research, especially with regard to ethics in neuro-ICT innovation contexts. Developing a global discourse ethics oriented toward matters of data governance, data sharing, and the ethical tensions between privacy and data protection, and open science and open data is another potentially productive strand of research. Such a global discourse ethics will have to find ways to incorporate local, national and regional sensitivities and particularities. In order to test whether our claim to generalisability can be upheld, it would be interesting to empirically test the transferability of our insights to other subject areas.

It is thus fair to say that much work remains to be done. However, drawing on 5 years of experience of developing and refining our dialogical approach to ethics and ethics support in the HBP we believe to have demonstrated that ethics does not have to be perceived as an imperialistic imposition. The dialogical approach presented here is likely to increase the knowledge base, be responsive to scientific developments and specific contexts and contribute to the promise of science to contribute to addressing the most pressing challenges humanity faces.

## Author Contributions

BS led the development of the ethics support work package and processes, wrote the first draft of the article and coordinated the contributions. SA worked on legal review and data protection and was part of the ethics check process. BF led the data governance and was involved in compliance management. MG led the ethics rapporteur programme and the PORE process. WK led the ethics compliance process. IU led the ethics awareness work. All authors worked closely together on the work described in the article. All have contributed to the text related to their area of expertise and contributed to the overall article, including through reviews and revision of earlier drafts.

## Conflict of Interest Statement

The authors declare that the research was conducted in the absence of any commercial or financial relationships that could be construed as a potential conflict of interest.

## Footnotes

^1^http://www.kavlifoundation.org/international-brain-initiative
